# Exercise-Induced Hyperhomocysteinemia Is Not Related to Oxidative Damage or Impaired Vascular Function in Amateur Middle-Aged Runners under Controlled Nutritional Intake

**DOI:** 10.3390/nu13093033

**Published:** 2021-08-30

**Authors:** Eduardo Iglesias-Gutiérrez, Ángela García-González, Ana Montero-Bravo, Antonio González-Medina, Judit Joglar, Cristina Tomás-Zapico, Benjamín Fernández-García, Manuel Fernández-Sanjurjo, David de Gonzalo-Calvo, Ángel Enrique Díaz-Martínez, Natalia Úbeda

**Affiliations:** 1 Departamento de Biología Funcional, Fisiología, Universidad de Oviedo, 33006 Oviedo, Spain; gestor1bio@uniovi.es (J.J.); tomascristina@uniovi.es (C.T.-Z.); manufsanjurjo@gmail.com (M.F.-S.); 2 Instituto de Investigación Sanitaria del Principado de Asturias (ISPA), 33011 Oviedo, Spain; fernandezbenjamin@uniovi.es; 3 Departamento de Ciencias Farmacéuticas y de la Salud, Facultad de Farmacia, Universidad San Pablo-CEU, CEU Universities, 28925 Alcorcón, Spain; angargon@ceu.es (Á.G.-G.); amontero.fcex@ceu.es (A.M.-B.); a.g.medina@hotmail.com (A.G.-M.); nubeda@ceu.es (N.Ú.); 4 Departamento de Morfología y Biología Celular, Universidad de Oviedo, 33006 Oviedo, Spain; 5 Investigación Traslacional en Medicina Respiratoria, Hospital Universitari Arnau de Vilanova y Santa María, IRBLleida, 25198 Lleida, Spain; dgonzalo@irblleida.cat; 6 CIBER de Enfermedades Respiratorias (CIBERES), Instituto de Salud Carlos III, 28029 Madrid, Spain; 7 Centro de Medicina del Deporte, Agencia Española de Protección de la Salud en el Deporte (AEPSAD), 28040 Madrid, Spain; enrique.diaz@aepsad.gob.es

**Keywords:** homocysteine, long-term acute exercise, oxidative stress, endothelial function

## Abstract

To determine the influence of different doses of maximal acute exercise on the kinetics of plasma homocysteine (tHcy) and its relationship with oxidative status and vascular function, nine recreational runners completed a 10 km race (10K) and a marathon (M). Blood samples were collected before (Basal), immediately post-exercise (Post0), and after 24 h (Post24). Nutritional intake was controlled at each sample point. A significant increase in tHcy was observed after both races, higher after M. Basal levels were recovered at Post24 after 10K, but remained elevated at Post 24 for M. A significant decrease in GSH/GSSG ratio was observed in Post0, especially marked after M. Furthermore, this increase in pro-oxidant status remained at Post24 only after M. Other oxidative status markers failed to confirm this exercise-induced pro-oxidant status except glutathione peroxidase activity that was lower in Post24 compared to Basal in 10K and in Post0 and Post24 in M. No statistical correlation was found between oxidative markers and tHcy. No significant changes were observed in the concentration of endothelial cell adhesion molecules (VCAM-1 and E-Selectin) and VEGF. In conclusion, tHcy increases in an exercise–dose–response fashion but is not related to endothelial dysfunction mediated by oxidative stress mechanisms.

## 1. Introduction

There is a close relationship between regular exercise and significant benefits in cardiovascular health: reduced risk of coronary heart disease, heart attack, and high blood pressure [1,2]. However, it is probable that the shape of the dose–response curve (exercise–benefits) may differ depending on the health outcome of interest and the baseline level of physical activity of the individual [3]. Therefore, the healthy exercise threshold needs to be explored because of the interactive effects of exercise volume, intensity, duration, and frequency, as well as the individual variability of response [4]. Usually, exercise recommendations have been formulated as the minimum amount of exercise of any nature that has a beneficial effect on health at different levels [5,6]. In consequence, it is also necessary to know the maximum amount of exercise that is associated with these benefits, without increasing the risk of other complications, at short or long term [7]. In this context, an increased risk for adverse cardiovascular events have been reported in ultraendurance athletes through increasing systemic inflammation [8,9,10,11,12,13,14], oxidative stress [14,15,16,17,18], or cardiac damage (mainly mediated by elevated levels of cardiac troponins and B-type natriuretic peptide) [19,20,21,22,23]. Furthermore, we have recently demonstrated that different exercise doses induced changes in circulating inflammamiRs and in miRNAs previously proposed as biomarkers of heart disease [11,24]. However, very few studies have informed of elevated plasma homocysteine (tHcy) in long–distance races [14,25,26].

Homocysteine is a non-protein sulphur-containing amino acid whose elevated plasma concentration constitutes an independent risk factor of cardiovascular disease (CVD), via endothelial dysfunction, oxidative stress mechanisms, and inflammatory vascular processes [27,28,29,30,31,32,33]. tHcy is strongly influenced by genetic and lifestyle factors such as diet, especially vitamins B_12_, B_6_ and folate [34]. However, the impact of exercise on tHcy is still unclear. A recent review [35] identifies that acute exercise induces an increase in tHcy. However, no consensus exists regarding chronic exercise, due to a large variety of exercise interventions, with different intensities, durations, and modes of exercise. Deminice et al. [36], in a meta-analysis, also reported that acute exercise increases tHcy independently of the duration or the intensity of the performed exercise, and that regular resistance training can decrease this parameter, though this was not observed after aerobic exercise training. On the other hand, combination of general physical activity and muscle-strengthening activities are associated with lower tHcy and there may be a dose–response relationship when combining both forms of exercise [37]. However, a recent study found no modifications in tHcy after a 12-week program of high-load or moderate-load resistance training [38].

We have previously reported that acute exercise at high (80% VO_2peak_) and low intensity (40% VO_2peak_) in healthy sedentary individuals causes a transient increase in tHcy proportional to the intensity of exercise, but not hyperhomocysteinemia [39]. Additionally, acute exercise of moderate-intensity, even at high frequeny of contraction (80 rpm of cycling cadence), has no negative effect on tHcy as an independent risk factor for CVD in sedentary people, when at least 400 kcal are spent during exercise and the nutritional status for folates and vitamin B_12_ were adequate [40]. However, this response could be different in active people. Some authors [35,41,42,43] showed that basal tHcy is lower in people with the greatest amount of daily physical activity. On the contrary, other studies [44,45] showed that athletes or physically active people have more elevated tHcy compared to sedentary controls or less active counterparts, with a 47% prevalence of hyperhomocysteinemia in the athlete group. It is possible, as we previously informed [39], that the higher basal tHcy reported in physically active people may be through the repeated, but transient, increase during each successive exercise bout and depends on the timing of post-exercise sample collection. This question still needs to be elucidated.

Therefore, based on this background, the objective of our study was to explore the influence of different doses of maximal aerobic exercise on the kinetics of tHcy in active male adults. Likewise, the relationship with endothelial function biomarkers and oxidative status will also be analysed.

## 2. Materials and Methods

### 2.1. Ethics Statement

The research was performed in accordance with the Declaration of Helsinki and all experimental procedures were approved by the Research Ethics Committee of the Principality of Asturias, Spain (reference 124/17). All participants gave written informed consent.

### 2.2. Experimental Design

Details on the sample, methods, and study design have been published elsewhere [11,24]. In short, volunteers were recruited from the members of MAPOMA Sports Association, a sports club open to amateur and professional runners, offering personalized training plans to their members. MAPOMA training plans for amateur runners are led by a team of sports and health professionals that promote a proper preparation for those wishing to participate in the popular Marathon of Madrid.

All male runners in the amateur training group (*n* = 35) were invited to an informative briefing. Only men were selected as subjects due to the stability of their hormonal status. A member of the research team presented the aims and methodology of the study and answered the questions of the potential participants, of whom 18 (51%) agreed to participate. The remainder (*n* = 17) showed interest in the study but were unable to participate for various reasons (family and professional commitments, travel, or injuries). Before participation, each volunteer underwent a thorough medical screening to determine eligibility. Several inclusion and exclusion criteria were also established. The inclusion criteria were as follows: (1) over 18 years of age; (2) officially registered for the Madrid marathon; (3) regular training (at least 50 km/wk); (4) previous participation in at least two marathons; and (5) written informed consent. The exclusion criteria were as follows: (1) any chronic disease; (2) body mass index (BMI) ≤30 kg/m^2^; (3) smokers or frequent passive smokers; (4) any dietary or pharmacological treatment during the study; and (5) electrocardiographic abnormalities. Although 14 volunteers fulfilled these criteria and were enrolled, only 9 participants completed the study. The remaining five participants were unable to finish at least one of the races or missed one of the blood extractions and their results were excluded from the final analysis.

Volunteers completed two races: a 10 km race (10K) and a marathon (M), separated by 1 mo. Although both trials involved the same type of exercise (endurance running), they represent distinct exercise doses, as they differ in terms of duration, relative intensity, and energy demands.

### 2.3. Baseline Evaluation

Body composition and aerobic capacity were assessed two weeks before the first race by using standardized methodology and protocols.

Body composition was assessed by two ISAK Level III certified anthropometrists. Height and body mass (BM) were measured using a combined medical scale (model 778, Seca Ltd., Hamburg, Germany; precision 0.1 cm for height and 0.1 kg for weight). The body mass index (BMI) was calculated from these measurements. The equation of Kyle et al. [46] was used to estimate percent body fat (%BF) based on the information obtained using a multifrequency bioimpedance system (Total Body Scan, Bio-logic Science Instruments España S. L., Barcelona, Spain). This equation was considered the most appropriate according to the position stand of the Spanish Group of Kinanthropometry [47].

A ramp protocol on a treadmill (LE-600 C, h/p/cosmos, Nussdorf-Traunstein, Germany) till volitional exhaustion was used to determine VO_2_max (Oxycon Pro, Jaeger, Hoechberg, Germany). The ratio of mean heart rate (HR) during each race (measured by personal HR monitors) and maximal heart rate (HR_max_) during the incremental protocol on the treadmill, was used to calculate the percent of HR_max_ (%HR_max_), to determine the individual exercise intensity during the different races [21].

During their visit to the laboratory, the volunteers were also interviewed to know their training history (years of training, number of marathon races previously finished, personal bests) and the volume of training in the last weeks (days per week, hours per day, and km per week).

### 2.4. Dietary Control

Participants were asked to keep a food diary for five consecutive days: two days before the first race, the race day, and the two following days. To minimize recording errors, volunteers received specific oral guidelines and detailed written instructions and two members of the research group were available during all the experimental period, by phone, to solve any doubts about the procedure. All foods and beverages were recorded using standard culinary measures; for information about food, supplements, snacks, and packed-foods, food labels were collected. Information about water and food intake during the races was also collected from the volunteers immediately after the end of each race. No limitations for the type or the amount of food or beverages consumed were established at any time during these five days of food recording, although participants were asked to abstain from caffeine and alcohol consumption for at least 24 h prior to each race.

Food records were carefully reviewed immediately after completion and subjects were contacted to clarify ambiguous information. Dietary records were analysed using a nutrient analysis software (DIAL^®^, Alce Ingeniería, Madrid, Spain).

In order to minimize the impact of food intake on results, volunteers were asked to repeat the same intake pattern on the days preceding and following the second race.

### 2.5. Blood Sampling

Blood samples were obtained by experienced technical staff, using standardized techniques and materials. Sampling points were the same for every race.

Subjects had their first blood sample taken about one hour before the race, in fasting state, and before starting warming-up (Basal). Another blood sample was drawn within 10 min after the cessation of exercise (Post0). In both cases, blood draws and the immediate processing of the samples were carried out in a field laboratory installed near the starting and finishing lines of the races, with the permission and cooperation of the organization staff. The following morning, subjects reported to the Clinical Laboratory at the Center for Sports Medicine at the same time as the previous day, after an overnight fast, and a new blood sample was taken (Post24).

The total volume of blood taken per race was less than 50 mL. Blood samples were collected in vacutainers (No Additive (Z), Becton Dickinson, Franklin Lakes, NJ, USA) and then centrifuged at 3000 rpm for 15 min at 4 °C. The plasma was immediately stored at −80 °C for later analysis.

### 2.6. Biochemical Determinations

#### 2.6.1. Homocysteine and Vitamins

tHcy and vitamin B_6_ (as pyridoxal-5-phosphate) concentration were determined by HPLC using a commercially available kit (Chromsystems Instruments & Chemicals GmbH, Munich, Germany) and fluorescent detection, where a derivatization process of the sample takes place. Once the sample is prepared, 50 µL are injected into the HPLC and fluorescence is measured at 385 nm excitation and 515 nm emission for tHcy and 320 nm excitation and 415 nm emission for vitamin B_6_.

Folate and vitamin B_12_ concentrations were measured using an ELECSYS system (Roche Diagnostics GmbH, Mannheim, Germany) based on an electrochemiluminescence immunoassay (ECLIA).

#### 2.6.2. Oxidative Stress

To evaluate oxidative stress, we determined in plasma: reduced glutathione (GSH); oxidized glutathione (GSSG); glutathione peroxidase (GPx), glutathione reductase (GR), total antioxidant capacity (TAC) and protein carbonyls as oxidative damage biomarker.

GSH and GSSG were determined by a commercial kit Bioxytech^®^ GSH/GSSG-412TM (OxisResearch, Portland, OR, USA) according to the manufacturer’s protocol.

We used a Cayman Chemical Glutathione Peroxidase Assay Kit (Cayman Chemical Company, Ann Arbor, MI, USA) that measures GPx activity indirectly by a coupled reaction with GR. The oxidation of NADPH to NADP+ is accompanied by a decrease in absorbance at 340 nm. The rate of decrease in the A340 is directly proportional to the GPx activity in the sample.

We also used a Cayman Chemical Glutathione Reductase Assay Kit (Cayman Chemical Company, USA) that measures GR activity by measuring the rate of NADPH oxidation. The oxidation of NADPH to NADP+ is accompanied by a decrease in absorbance at 340 nm. Since GR is present at rate limiting concentrations, the rate of decrease in the A340 is directly proportional to the GR activity in the sample.

Total antioxidant capacity (TAC): TAC was measured with a Potential Anti-Oxidant (PAO) assay kit (JaICA Nikken SEIL Co., Ltd., Fukuroi, Japan) which TAC of samples can be detected using the reduction in cupric ion (Cu^2+^ to Cu^+^) in a colorimetric assay: 480–492 nm.

Absorbance signals were collected using the Beckman Coulter DU-650 Spectrophotometer (Brea, CA, USA).

Protein carbonyls were measured using a commercial enzyme-linked immunosorbent assay (ELISA) (Zentech PC Test, Protein Carbonyl Enzyme Immuno-Assay Kit; Zenith Technologies, Dunedin, New Zealand).

#### 2.6.3. Endothelial Function

Endothelial function was evaluated by the determination of soluble endothelial adhesion molecules: Vascular cell adhesion molecule-1 (VCAM-1) and E-selectin, as well as vascular endothelial growth factor (VEGF).

VCAM-1 and VEGF were determined by commercial enzyme-linked immunosorbent assay (ELISA) (Abcam, Cambridge, UK). The E-selectin levels were measured using Randox Evidence Investigator analyser (Randox Laboratories, Crumlin, UK).

### 2.7. Statistical Analysis

Descriptive statistics were used to characterize study population and to describe the studied parameters. Normality was determined using the Kolmogorov–Smirnov test. In light of the results obtained, descriptive values are presented as means ± SD. Student’s *t* test for related samples were used to compare parameters between races (10K and M). One-way ANOVA for repeated measures with Bonferroni correction was used to compare parameters between sampling time points (Basal, Post0, and Post24). Fold change with respect to the Basal sample was determined to quantify the magnitude of the response. To check the relationship among the different variables at each time point, a full correlation analysis was made by using Pearson correlation coefficient and linear regression analysis. The level of significance was set at *p* < 0.05 for all statistical tests. Descriptive and analytical statistical analyses were performed using IBM SPSS Statistics, version 24.0 (Somers, NY, USA).

## 3. Results

### 3.1. Physical Characteristics and Training Profile

Anthropometric data, training practices and training history of the participants have already been published elsewhere [11]. The overall profile of the participants can be interpreted as the one of a middle-aged man, experienced amateur runner with moderately high workloads.

### 3.2. tHcy Kinetics

**Figure 1:**
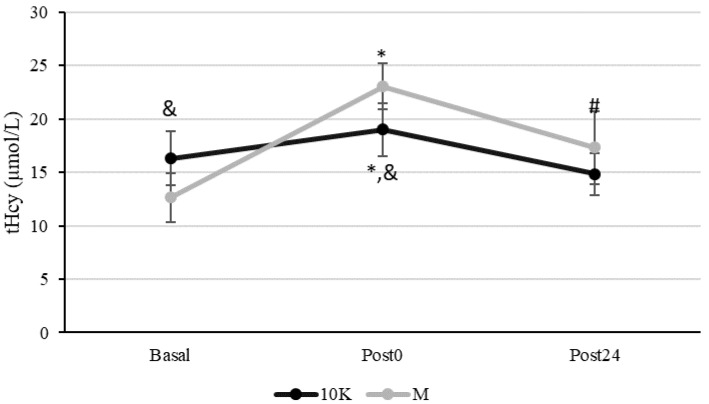
Kinetics of plasma homocysteine concentration (tHcy, µmol/L) in response to a 10 km race (10K, black line) and a marathon (M, grey line). Basal: sample collected within 1 h before each race; Post0: sample collected within 10 min after the race; Post24: sample collected 24 h after Basal. * Significantly different (*p* < 0.05) from Basal and Post24; # Significantly different (*p* < 0.05) from Basal. & Significantly different (*p* < 0.05) from Marathon (M). Data are presented as mean ± standard deviation. Sample size, N = 9.

Figure 1 shows the tHcy kinetics in response to 10K and M. tHcy basal levels were statistically higher before 10K compared to M. It is remarkable that the basal concentration of this amino acid was very close to the higher limit of normo-levels or even above it (15 µmol/L).

tHcy increased significantly at Post0 both for 10K and M, being this increase significantly higher after M (16% vs. 82%, *p* < 0.005). Furthermore, tHcy recovered Basal levels at Post24 after 10K but not after M, remaining significantly higher than Basal (*p* = 0.001). Higher tHcy levels were observed for M at Post0 (*p* = 0.005) than 10K and a similar tendency was also observed at Post24 (*p* = 0.066).

### 3.3. Oxidative Status

**Figure 2:**
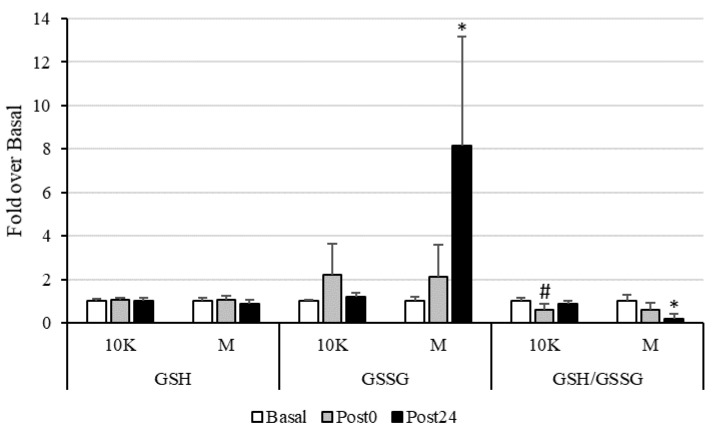
Reduced (GSH) and oxidized (GSSG) glutathione concentrations, as well as GSH/GSSG ratio, expressed as fold over Basal, trough the different races and timepoints. 10K: 10 km race; M: Marathon race. Basal: sample collected within 1 h before each race; Post0: sample collected within 10 min after the race; Post24: sample collected 24 h after Basal. * Significantly different (*p* < 0.05) from Basal and Post0; # Significantly different (*p* < 0.05) from Basal and Post24. Data are presented as mean ± standard deviation. Sample size, N = 9.

As seen in Figure 2, an increase in pro-oxidant status was observed after exercise. This was particularly evident after M, with a significant increase in GSSG and a decrease in GSH/GSSG ratio at Post24 compared to Basal and Post0. Regarding 10K, an acute significant decrease in GSH/GSSG ratio was observed at Post0, recovering Basal levels at Post24.

**Figure 3:**
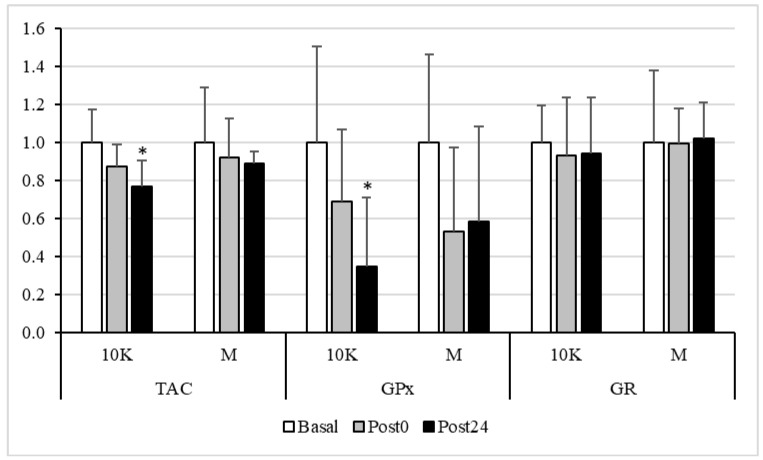
Total antioxidant capacity (TAC), Glutathione Peroxidase (GPx) and Glutathione reductase (GR) activities, expressed as fold over Basal, trough the different races and timepoints. 10K: 10 km race; M: Marathon race. Basal: sample collected within 1 h before each race; Post0: sample collected within 10 min after the race; Post24: sample collected 24 h after Basal. * Significantly different (*p* < 0.05) from Basal and Post0. Data are presented as mean ± standard deviation. Sample size, N = 9.

The GPx activity (Figure 3) was also altered by exercise in the 10K race, showing a decrease at Post24. In M, the decrease was seen earlier (although not significative), at Post0, and remained at Post24. In line with the above results, TAC only decreased in response to 10K at Post24. On the other hand, when studying the changes in the activity of the GR, no differences were observed between races, at any sampling point.

**Figure 4:**
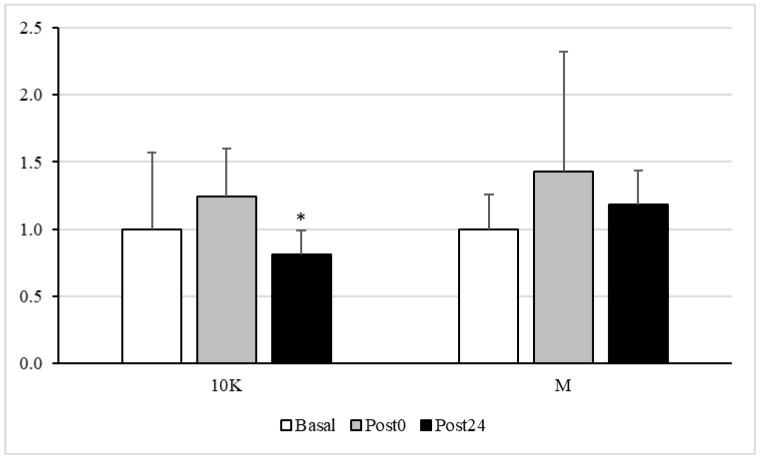
Plasma protein carbonyl, expressed as fold over Basal, trough the different races and timepoints. 10K: 10 km race; M: Marathon race. Basal: sample collected within 1 h before each race; Post0: sample collected within 10 min after the race; Post24: sample collected 24 h after Basal. * Significantly different (*p* < 0.05) from Basal and Post0. Data are presented as mean ± standard deviation. Sample size, N = 9.

Figure 4 shows plasma protein carbonyls response to different doses of acute exercise. In response to 10K, this parameter decreased significantly at Post24 vs. Basal and Post0.

No statistical correlation was found between tHcy response to 10K and M and any of the oxidative stress markers measured at any timepoint.

### 3.4. Endothelial Function

**Figure 5:**
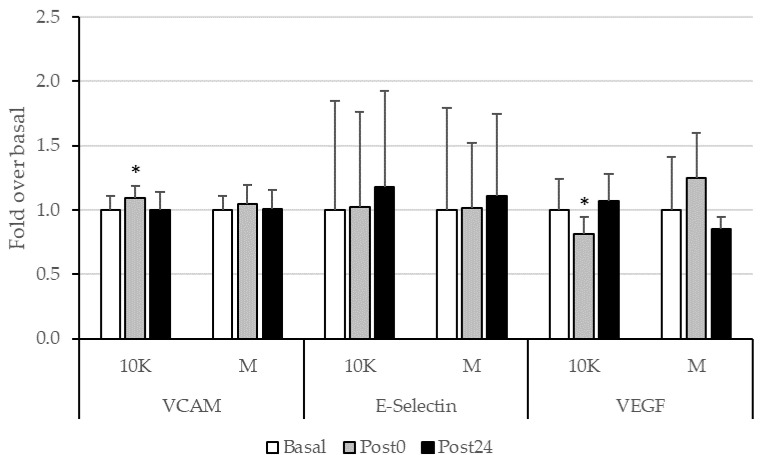
Adhesion molecules (VCAM-1 and E-Selectin) and VEGF, expressed as fold over Basal, trough the different races and timepoints. 10K: 10 km race; M: Marathon race. Basal: sample collected within 1 h before each race; Post0: sample collected within 10 min after the race; Post24: sample collected 24 h after Basal. * Significantly different (*p* < 0.05) from Basal. Data are presented as mean ± standard deviation. Sample size, N = 9.

We observed minimal effect of different doses of acute exercise on the concentration of the adhesion molecules VCAM-1 and E-Selectin (Figure 5). Nevertheless, it is remarkable the observed significant decrease in the VEGF levels at Post0 for 10K.

No significative relationships were neither found between tHcy and the endothelial function parameters measured.

### 3.5. Vitamin Levels and Dietary Intake

The levels of the vitamins involved in tHcy metabolism (folate, vitamin B_6_ and vitamin B_12_) are shown in Table 1. They were in normal ranges of concentrations, in all sampling points, in all races.

A negative correlation was found between tHcy levels and those of vitamin B_6_ in 10K Post0 (r −0.738, *p* = 0.037) and in M post24 (r −0.874, *p* = 0,005). The same negative correlation was seen between Hcy and folates concentration in M at post24 (r −0.803 *p* = 0.009).

Table 2 shows the average energy, macronutrient, and homocysteine-related vitamin intake during the pericompetitive period.

The diet in the pericompetitive period was, in general, adequate to accomplish the targets for energy and vitamin intake for the correspondent age group, considering they are active [49]. The macronutrient distribution of energy was also adequate when compared to the Nutritional Goals for the Spanish population [49].

## 4. Discussion

Although the exercise responses and adaptations that are beneficial to cardiovascular health are dose-dependent [50], adverse consequences of prolonged acute aerobic exercise have also been described, suggesting that a maximum safe dose potentially exists [51]. In this sense, it raises some concern the increasing participation of amateur runners in long-distance races. In 2016, there were approximately 2.5 million participants in marathons and half-marathons in the USA, compared to less than 1 million in 2000 [52]. This study approximates for the first time to the kinetics and metabolism of tHcy, an independent risk factor for CVD, in response to different doses of acute aerobic exercise in active people. The response of endothelial function and oxidative stress biomarkers, relevant in the pathophysiology of CVD mediated by tHcy [53], were also analysed.

In this study, we have shown an acute increase in tHcy post exercise, being more accused after M (82% vs. 16%) compared to 10K and remaining further in time. Several authors have also found an elevation in tHcy after acute exercise [35,36], some of them in response to long distance races, such as marathon, triathlon, or ultramarathon [14,25,26]. Interestingly, we have observed a pronounced acute increase, reaching hyperhomocysteinemia values, what was not observed in other studies. In the fasting state, tHcy normally ranges from 5 to 15 mmol/L [54]. Thus, hyperhomocysteinemia has been defined as concentrations >15 mmol/L [55]. It has been estimated that a 2.5 µM rise in tHcy is associated with a 10% increase in CVD risk [56]. Moreover, it was shown that increased tHcy (above 20 µM) is associated with a nine-fold increase in myocardial infarction risk and also with subsequent stroke risk, compared to concentrations below 9 µM [57]. The subjects who participated in this study had tHcy levels ranging from 12.7 to 23.0 µmol/L, values that are higher than those shown in other studies with population groups similar in age, gender, and lifestyle [14,26,27,58]. Nevertheless, these data are consistent with observations from other studies that determined a higher tHcy at rest in physically active compared to sedentary people [45,46]. Furthermore, according to our previous studies [39,40], the maximal tHcy (Cmax) in response to an acute bout of exercise occurs during exercise, matched with a particular energy expenditure. Furthermore, the magnitude of Cmax is irrespective of exercise intensity, but depends on the duration of the activity. In this sense, the increase observed in tHcy may have been even higher during the exercise bouts, particularly during M. Since this increase persist considerably over time (more than 24 h), we should not discard that it may be causing damage at the endothelial level through oxidative or other mechanisms [27,33].

Hyperhomocysteinemia can increase CVD through various mechanisms, among which increasing oxidative stress and endothelial dysfunction play a preeminent role [29,32,33]. We have measured a panel of parameters related to these mechanisms at the same time-points that tHcy, in order to study its relationship with endothelial dysfunction through oxidative stress mechanisms in the response to different doses of aerobic exercise.

Glutathione is considered a marker widely used as an indicator of redox status, and homocysteine is also metabolically related to glutathione synthesis. The antioxidant tripeptide glutathione is synthesized from cysteine, which in turn is synthesized from homocysteine by the transsulfuration pathway, which requires the presence of vitamin B_6_ for the proper functioning of the enzymes involved, cystathionine β-synthase and cystathionine γ-lyase [58]. We observed a significant decrease in the ratio GSH/GSSG at Post0 after 10K related to a non-significant 120% increase in GSSG at this timepoint, recovering basal concentrations after 24 h. Furthermore, plasma vitamin B_6_ concentration decreased a 17% at Post0 and a 25% at Post24 after 10K. Although this decrease was not statistically significant, it may be indicating an increase in transsulfuration pathway to synthesize GSH, which is rapidly used as antioxidant molecule giving rise to GSSG. The same pattern was observed in response to M, although the magnitude of variation was higher, and these parameters remained significantly altered after 24 h (except for the decrease in plasma vitamin B_6_).

No significant difference in GR activity was observed between the different timepoints. However, the activity of GPx decreased after both exercise doses, being significantly different from Basal at Post24 in response to 10K. It is reasonable to think that the activity of this enzyme should be maximal during exercise, catalysing the oxidation of glutathione to reduce some ROS and, thus, preventing the oxidation of macromolecules, which, therefore, could be reduced post-exercise. In fact, a significant decrease in oxidative protein damage was observed at Post24 after 10K.

Furthermore, no statistical correlations were found between tHcy and these oxidative stress markers. Therefore, an increase in pro-oxidant status due to exercise, was observed in this study, but not related to tHcy increase. Several studies found an increase in oxidative stress in long-term races [14,15,16,17,18,59,60,61,62,63]. According to de Lucas et al. [17] ultra-endurance events provoked marked oxidative stress, likely by increasing oxidative metabolism (increased oxidative mitochondrial function), what would correspond in our study with the highest level of oxidative stress observed in response to M. This might be advantageous during prolonged exercise, mainly for efficient substrate oxidation at the mitochondrial level, even when tissue damage is induced. Furthermore, although acute exercise leads to increased oxidative stress, this same stimulus is necessary to allow an up-regulation in endogenous antioxidant defences (hormesis) [64,65]. However, ultra-endurance-race-induced oxidative stress, persists for one calendar month depending on the specific biomarker examined. In this sense, Turner et al. observed that glutathione, the marker with the highest percentage of variation due to exercise in our study, remained depleted to approximately one-third of prerace values 28 d after a 233 km running event [16]. We did not examine these markers beyond 24 h, but the 10K and M were separated by a month and these parameters showed normal baseline values at baseline in all of them, indicating a total recovery of oxidative status.

In addition, it has been suggested the existence of a process of adaptation to oxidative stress as a result of training, so that trained subjects would have greater resistance to oxidative damage after performing acute exercise. This hypothesis argues that during long-term training the body adapts to stress and regulates redox homeostasis, concluding that exercise training seems to induce an antioxidant effect [66]. In this sense, some authors demonstrated that the group with the low exercise-induced oxidative stress exhibited the lowest improvements in a battery of classic adaptations to endurance training (VO_2 max_, time trial and Wingate test) as well as in a set of redox biomarkers (oxidative stress biomarkers and antioxidants), compared to the high and moderate oxidative stress groups [67]. Therefore, training is associated with a chronic increase in antioxidant enzyme activity, being able to provide protection against the increase in exercise-induced oxidative stress. Repeated exposure to a certain stress factor, in this case exercise, implies a decrease in ROS production. This could explain why we did not find an increase in oxidative damage to macromolecules after acute exercise, especially in response to M, as previously described in similar studies [68,69,70]. However, whether these changes have long-term negative effects in the organism needs further investigation.

On the other hand, as already explained, both prolonged exercise and homocysteine can cause endothelial damage. We measured some biomarkers of inflammation or vascular dysfunction, but we did not find important variations with physiological or pathological significance in any of the sampling points in the two races. In the same way, they did not correlate with tHcy. Some studies found increases VCAM-1 and E-selectin in endurance sports [71,72,73,74,75] related to intensity [71] and duration [73]. In our study, the absence of alteration in these parameters could be resembling an adaptation to training. Furthermore, VCAM-1 levels have been described to be positively correlated with running speed [71], which could be in the basis of the higher levels observed after 10K compared to M.

The plasma VEGF concentration in the subjects was also analyzed before and after the different races, resulting in a decrease Post0 after 10K race, although with no relevant variations in the plasma levels of this marker. Several studies show that VEGF blood levels decreases with exercise [76,77] and others show increases [78,79,80,81] independent of training status [79,80]. Again, it is observed that variance in exercise-induced increases in inflammatory parameters in response to prolonged endurance exercise was characterized by exercise metabolic demand and cardiorespiratory fitness measures in endurance trained athletes [80,81].

tHcy was not related to oxidative parameters but is strongly dependent of some B-vitamin status. Vitamins involved in homocysteine metabolism (folate, vitamin B_6_ and vitamin B_12_) were in adequate serum concentrations and showed the typical response after acute exercise, as we previously showed [39,40]. We found negative correlation between tHcy and vitamin B_6_ and folate. However, despite normal serum vitamin concentration and adequate diet, elevation of tHcy occurred, so the increase can only be explained as a physiological response to exercise, maybe related to changes in glomerular filtration and plasma clearance [82]. So, in light of our results, adequate folate, vitamin B_6_ and vitamin B_12_ intake could be especially important for people that are usually active, and who would be exposed to transient but enduring increases in tHcy.

Our study has several strengths and limitations. The strengths of our study are the meticulous experimental design and the strict control and characterization of the volunteers, including dietary habits. The repeated measures-nature of this study, barely used in the literature available, reduced the variability in the response to the different conditions tested (exercise doses), strengthening the statistical analysis. We recruited amateur athletes, who represent a great proportion of participants in endurance events. We encouraged the volunteers not to alter their usual training schedule or food habits; therefore, the response to exercise was explored in “real-life” settings. Some limitations of the present study should be noted. First, the strict inclusion criteria and the invasive nature of the study limited the possibility of recruiting a larger number of subjects. However, as mentioned before, in the repeated-measures design of the present study, the same 9 subjects participated in two different acute exercise bouts (10K and M) and provided three samples before and after each event, which led us to analyse more than 50 samples. Second, generalization of results is limited by the characteristics of the study subjects: middle-aged, physically active males, without cardiovascular risk factors. Thus, the conclusions have been written accordingly. Finally, although the aim of this study focused on tHcy, the analysis of other cardiometabolic risk factors could have also been of interest. However, considering the plethora of cardiometabolic risk factors, the parameters selected could be biased by subjective selection. Furthermore, considering that each parameter could show different plasma appearance-clearance kinetics, the experimental protocol designed could not account for all this heterogeneous response.

## 5. Conclusions

We have observed that acute exercise-induced hyperhomocysteinemia in trained individuals is dose-dependent, but it was not related to oxidative damage to macromolecules or endothelial dysfunction. Therefore, the present investigation did not specifically evidence a contribution of tHcy in promoting metabolic changes that can lead to adverse cardiovascular events in amateur runners after different doses of acute exercise. Nevertheless, data suggest that an appropriate training and recovery time, as well as adequate diet must be carefully planned in order to guarantee low tHcy in response to acute exercise.

## Figures and Tables

**Table 1:** Plasma levels of the main vitamins involved in homocysteine metabolism in response to different doses of acute exercise.

Plasma Vitamin Concentration	Timepoint	10K	M
**Vitamin B_12_ (pmol/L)**	Basal	500.5 ± 86.1 ^1,a^	527.1 ± 75.5 ^1,a,b^
	Post0	547.1 ± 106.7 ^2,a^	584.2 ± 74.1 ^2,a,b^
	Post24	472.0 ± 99.5 ^3^	477.4 ± 72.1 ^3^
**Vitamin B_6_ (nmol/L)**	Basal	84.7 ± 52.9	111.4 ± 70.3
	Post0	70.2 ± 36.5	66.1 ± 25.9
	Post24	63.6 ± 28.3	95.9 ± 56.0
**Folate (nmol/L)**	Basal	7.6 ± 1.9 ^1,a^	10.9 ± 3.4 ^1,b^
	Post0	10.1 ± 3.0 ^2,3^	11.2 ± 2.8 ^1,2^
	Post24	8.1 ± 2.7 ^1^	8.9 ± 2.3^.2^

10K: 10km race; M: Marathon race. Basal: sample collected within 1 h before each race; Post0: sample collected within 10 min after the race; Post24: sample collected 24 h after Basal. Different letter superscripts show statistical differences (*p* < 0.05) within races; different number superscripts show statistical differences (*p* < 0.05) within sampling points. Data are presented as mean ± standard deviation. Sample size, N = 9.

**Table 2:** Nutritional intake in the pericompetitive period, recommended dietary intakes, and nutritional goals for the Spanish population.

Nutrient	Mean ± SD	Targets [48,49]
**Energy intake (kcal/day)**	2671 ± 627	-
**Carbohydrates (% of Energy)**	45 ± 3	50–55
**Lipids (% of Energy)**	33 ± 5	30–35
**Proteins (g/day)**	90 ± 19	54
**Folates (µg)**	350 ± 128	400
**Vitamin B_12_ (µg)**	6.3 ± 2.9	2
**Vitamin B_6_ (mg)**	2.4 ± 1	1.8

SD: Standard deviation. Sample size, N = 9.

## Data Availability

The data presented in this study are available on request from the corresponding author.
